# Multiple Interactions between Peroxisome Proliferators-Activated Receptors and the Ubiquitin-Proteasome System and Implications for Cancer Pathogenesis

**DOI:** 10.1155/2008/195065

**Published:** 2008-06-08

**Authors:** Davide Genini, Giuseppina M. Carbone, Carlo V. Catapano

**Affiliations:** Laboratory of Experimental Oncology, Oncology Institute of Southern Switzerland (IOSI), Via Vela 6, 6500 Bellinzona, Switzerland

## Abstract

The peroxisome proliferator-activated receptors (PPAR) *α*, *β*/*δ*, and *γ* are ligand-activated nuclear receptors involved in a number of physiological processes, including lipid and glucose homeostasis, inflammation, cell growth, differentiation, and death. PPAR agonists are used in the treatment of human diseases, like type 2 diabetes and dyslipidemia, and PPARs appear as promising therapeutic targets in other conditions, including cancer. A better understanding of the functions and regulation of PPARs in normal and pathological processes is of primary importance to devise appropriate therapeutic strategies. The ubiquitin-proteasome system (UPS) plays an important role in controlling level and activity of many nuclear receptors and transcription factors. PPARs are subjected to UPS-dependent regulation. Interestingly, the three PPAR isotypes are differentially regulated by the UPS in response to ligand-dependent activation, a phenomenon that may be intrinsically connected to their distinct cellular functions and behaviors. In addition to their effects ongene expression, PPARs appear to affect protein levels and downstream pathways also by modulating the activity of the UPS in target-specific manners. Here we review the current knowledge of the interactions between the UPS and PPARs in light of the potential implications for their effects on cell fate and tumorigenesis.

## 1. INTRODUCTION

Despite the everyday progress in understanding the genetic and molecular bases of cancer, this disease still strikes millions of people worldwide. The quest for new targets and more effective therapeutics is currently a major driving force in cancer research. Multiple mutations that affect critical cellular pathways lead to uncontrolled proliferation, increased survival, and block of differentiation in cancer cells [1]. Several cellular pathways (e.g., cell surface receptors, signal transduction pathways, apoptosis, angiogenesis, transcription, chromatin regulation, and proteasome-mediated degradation) have provided relevant targets and opportunities for development of clinically useful therapeutics [1]. Unfortunately, targeting each of these major pathways individually may not be sufficient. Extensive cross-talks occur between regulatory pathways and it is not unlikely that the same proteins play multiple roles in different processes. The peroxisome proliferator-activated receptor (PPAR) subfamily of nuclear receptors may represent a prime example of proteins interacting with multiple cellular pathways and exerting diverse and sometime apparently contrasting functions. Here, we review how PPARs interact with the ubiquitin-proteasome system (UPS), which is the major cellular system responsible for protein turnover, and how these two systems might reciprocally affect each other activity and functions.

## 2. THE UBIQUITIN-PROTEASOME SYSTEM

Ubiquitin is a 76-amino acid polypeptide that is post-transcriptionally linked to proteins via a covalent linkage to one or multiple lysine residues [2]. Several proteins including cell surface receptors, cell cycle regulators, and transcription factors are ubiquitinated and protein ubiquitination affects many cellular processes including proliferation, cell cycle progression, DNA damage repair, and cell death [2]. Ubiquitination is a regulatory signal that affects the fate and function of proteins. Ubiquitination regulates mainly protein turnover directing ubiquitinated proteins to proteasome-mediated proteolysis. Other nonproteolytic functions, like control of protein-protein interactions, cellular localization, and catalytic activity, are emerging [2]. The proteasome is a multicatalytic complex that comprises a 20S core with proteolytic activity and a 19S subunit that recognizes poly-ubiquitinated proteins, unfolds them, and passes into the 20S catalytic core for degradation. Ubiquitination is catalyzed by three types of enzymes, called E1, E2, and E3 [2, 3]. Ubiquitin is first activated by an E1 ubiquitin-activating enzyme in an ATP-dependent reaction. The activated ubiquitin is then transferred to an E2 ubiquitin-conjugating protein (UBC). Finally, E3 ubiquitin-ligases, which are the most critical enzymes in the process, catalyze the transfer and covalent attachments of the activated ubiquitin to the target protein. In human cells, a single E1 and about 60 E2 enzymes have been identified, while there are approximately a thousand E3 enzymes, which ensure a high degree of substrate specificity to the system [2, 3]. E3 enzymes are split in two major subfamilies: the Ring-H2 and the HECT domain proteins. The human genome contains also more than 70 deubiquitinating enzymes (DUBs) that remove ubiquitin chains from ubiquitinated proteins and can rescue them from proteasomal degradation [4].

Protein ubiquitination is a highly dynamic process and ubiquitination-deubiquitination cycles can serve to rapidly modulate protein level and function [4]. Ubiquitin and proteasomal components play an important role in transcription [5, 6]. Ubiquitin ligases and proteasomal subunits are present as integral components of transcription regulatory complexes [5, 6]. Histones, the main component of chromatin, are ubiquitinated and the process affects chromatin remodeling and transcription [6, 7]. RNA polymerase II is also directly regulated by ubiquitination [6, 8]. Moreover, the UPS regulates the abundance, activity, and subcellular localization of many transcription factors [5, 6]. Transcription factors are ubiquitinated and degraded by the proteasome and, paradoxically, the process is often essential for their transactivating ability [6]. In fact, transcription activation and degradation domains of transcription factors often overlap [6]. In addition, mono-ubiquitination (i.e., addition of single ubiquitin tag to a protein) can act as a post-translational modification that modulates activity of transcription factors and regulates transcription efficiency by nonproteolytic mechanisms [6]. Degradation of inhibitors of transcription factors is also often required to release active transcription factors. For example, activation of the transcription factor NF-*κ*B is controlled by a signaling cascade based on multiple ubiquitination and proteasome-dependent events [6].

Alterations of the UPS are frequent in cancer. They are mainly due to loss or gain of function of specific components of the UPS and alterations of UPS substrates, like oncogene and tumor suppressor gene products, which become less or more susceptible to proteasomal-dependent degradation [9]. Tumor suppressor proteins are often the targets of UPS alterations. The human papillomavirus (HPV), a cause of cervical cancer, encodes two oncogenic proteins, E6 and E7. These viral proteins promote degradation of the tumor suppressor p53 via ubiquitination by the E6-associated protein (E6-AP) E3 ubiquitin ligase [10]. HDM2 is another E3 ubiquitin ligase that targets p53 to proteosomal degradation [11]. Aberrant expression of HDM2 is found in many human cancers [12]. Single nucleotide polymorphism in the HDM2 promoter leading to HDM2 overexpression has been recently associated to the development of sporadic and hereditary cancers [13]. The E3 ubiquitin ligase Skp2 is responsible for ubiquitination of the cell cycle inhibitor and tumor suppressor p27 [14]. Skp2 overexpression is observed in cancer cells leading to degradation and inactivation of this tumor suppressor protein [15]. Oncogenic proteins are also affected by alterations of UPS components. The E3 ubiquitin ligase encoded by the von Hippel-Lindau gene (pVHL) mediates the ubiquitination and degradation of the hypoxia-inducible transcription factor HIF-1*α* [16, 17]. Mutations in pVHL gene predispose patients to renal cell carcinoma and other cancers. In these tumors, the level of HIF-1*α* is increased resulting in a potent oncogenic and angiogenic stimulus.

Due to the unique mechanism of cleavage at the proteolytic active sites, selective proteasome inhibitors have been synthesized and some, like bortezomib (Velcalde, PS341), have undergone clinical evaluation as anticancer agents [18]. Bortezomib is a peptide boronate proteasome inhibitor that blocks the chymotryptic activity of the 26S proteasome [18]. The anticancer effect of bortezomib is likely to be achieved through its inhibitory effects on protein degradation and modulation of important cellular pathways, including inhibition of the NF-*κ*B pathway [18]. Bortezomib is currently approved for clinical use for treatment of multiple myeloma. Clinical trials with bortezomib and second generation proteasome inhibitors as single agents or in combination with other chemotherapeutic agents are ongoing in various tumor types [18].

## 3. PEROXISOME PROLIFERATOR-ACTIVATED RECEPTORS

PPARs emerged in the nineties as nuclear receptors regulating transcription of genes involved in metabolic processes like lipid and glucose homeostasis [19, 20]. Later, PPARs have found to be implicated in many physiological and pathological processes [20]. PPARs belong to the nuclear hormone receptor super-family, which is one of the largest families of transcriptional regulators in the human genome with more than 40 distinct nuclear receptors [21]. Nuclear receptors bind small lipophilic molecules, such as steroid hormones, vitamins, and fatty acid derivatives, and function as ligand-activated transcription factors, interacting with specific DNA sequences (i.e., hormone response elements, HRE) in target genes and stimulating their transcription [21]. Thus, nuclear receptors provide a direct link between small lipophilic signaling molecules present in the cells or their environment and the cellular transcriptional machinery, turning on specific subsets of genes containing the appropriate HRE and inducing complex cellular responses [21, 22]. The nuclear receptor super-family includes the steroid hormone receptors (i.e., estrogen, progesterone, androgen, and glucocorticoid receptors) and receptors for nonsteroidal hormones [21–23]. The latter include the PPARs, vitamin D (VDR), and retinoic acid (RAR) receptors [21, 23]. The ligands of most nonsteroidal receptors are dietary fatty acids or generated locally by lipid metabolism within the target cell or tissue, while steroid and thyroid hormones are produced by distant endocrine organs and released in the blood [21, 22].

Nuclear receptors exhibit a characteristic modular structure comprising an N-terminal domain with the ligand-independent activation function domain (AF-1), a DNA binding domain (DBD), and a C-terminal domain containing the ligand binding (LBD), and the ligand-dependent transactivation (AF-2) domain [23]. The DBD contains two zinc finger modules and determines the DNA binding specificity of the receptors. The LBD is involved in homo- and heterodimerization and interaction with cofactors [23, 24]. The structure of the LBD is highly conserved among nuclear receptors, comprising a large hydrophobic cavity that accommodates the ligand. Variations of the size and shape of the ligand binding pocket ensure ligand specificity among receptors [23, 24]. Ligand-binding induces a conformational remodeling of the LBD that exposes surfaces required for interaction with coactivators and affects the affinity of the receptors for corepressors [23, 24]. The nonsteroidal receptors are found primarily in the cell nucleus and are bound to HRE as heterodimers with the retinoic X receptor (RXR) [19, 23]. These receptors can affect both positively and negatively transcription of target genes with the LBD mediating alternatively transcriptional activation or repression, although the mechanisms of transrepression by PPARs are still poorly understood [25]. Transcriptional repression is due to recruitment of corepressors, like NCoR/SMART, by the unliganded and DNA-bound receptor and formation of multiprotein complexes containing histone deacetylases and other chromatin remodeling enzymes [23, 25]. In the presence of ligands, corepressor complexes are released and replaced by coactivators, like SRC1 and CBP-p300, thus switching on transcription [23, 25]. Transcriptional activation is associated with histone modifications, chromatin remodeling, and assembly of the transcription initiation complex. Thus, transcriptional activation and repression by nuclear receptors are very dynamic processes involving the formation of protein complexes in which multiple coactivators and corepressors need to be rapidly exchanged [25–27]. The UPS is perhaps the major system controlling the assembly and turnover of these regulatory complexes ensuring their timely interaction with the transcriptional machinery [26]. Ubiquitin and proteasome components are associated with corepressor and coactivator complexes recruited by nuclear receptors [25, 26]. Most nuclear receptors, including thyroid hormone, estrogen, glucocorticoids receptor, RAR, and RXR receptors, as well as coactivators, corepressors, and general components of the transcription machinery are ubiquitinated and degraded by the proteasome [26, 28].

**Figure 1:**
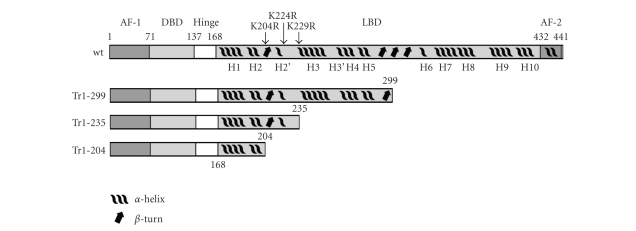
Domain structure of PPAR*δ* and truncated forms of the receptor. AF-1, N-terminal ligand-independent activation function 1 (aa 1–70). DBD, DNA binding domain (aa 71–136). Hinge, flexible hinge region (aa 137–167). LBD, ligand-binding domain (aa 168–431). AF-2, C-terminal ligand-dependent activation function-2 (aa 432–441). The position of the mutations (K204R, K224R, and K229R) introduced in the region 204–235 is shown.

PPARs have the typical modular structure of the nuclear hormone receptors with a poorly characterized N-terminal domain with putative ligand-independent AF-1 function, a central DNA-binding domain (DBD), and a C-terminal ligand binding (LBD) and ligand-dependent AF-2 domain (Figure 1) [19, 23]. However, despite the high sequence and structural homology, the three PPAR isotypes have distinct ligand specificity, functions, and behaviors [19, 20]. PPAR*α* is a key regulator of energy homeostasis and plays a major role in lipid metabolism and glucogenesis. PPAR*α* is expressed in tissues with significant fatty acid and cholesterol catabolism, like brown adipose tissue, liver, kidney, intestine, heart, and skeletal muscle [29]. PPAR*γ* exists in two isoforms (*γ*1 and *γ*2) that differ only at the N-terminus. PPAR*γ*2 is present at high levels in adipose tissue, whereas PPAR*γ*1 expression is broader and is present in gut, brain, vascular cells, immune cells, and retina [30]. PPAR*γ* plays a role in adipocyte differentiation, glucose metabolism, and lipid homeostasis, and participates in monocyte/macrophage differentiation [30]. Moreover, PPAR*γ* influences fatty acid storage in the adipose tissue and is implicated in insulin resistance and atherosclerosis [30]. PPAR*δ* is ubiquitously expressed with high levels in colon, skin, and brain [20]. PPAR*δ* also functions in processes linked to lipid metabolism, like fatty acid catabolism, cholesterol efflux, lipid uptake in macrophages, and preadipocyte differentiation [31]. This nuclear receptor plays also a role in placental and gut development, embryo implantation, tissue injury, and wound healing [20, 32].

PPARs possess a broad ligand-binding cavity that allows binding of a wide range of synthetic and natural lipophilic compounds [19]. Medium- and long-chain unsaturated fatty acids (e.g., linoleic acid), conjugated and oxidized fatty acids (e.g., phytanic acid), and eicosanoids bind to PPAR*α* [19]. Fibrates, like bezafibrate, fenofibrate, and clofibrate, which are used for the treatment of dislipidemias and cardiovascular diseases, are selective PPAR*α* agonists [29]. PPAR*γ* binds to long-chain fatty acids, prostaglandin J_2_ (PG J_2_), and other eicosanoids [19]. Synthetic PPAR*γ* agonists, such as pioglitazone and rosiglitazone, are insulin sensitizers used to treat type 2 diabetes [30]. PPAR*δ* has high affinity for prostaglandin I_2_ (PGI_2_), fatty acids, and synthetic compounds [19, 31].

Beside their metabolic functions, PPARs have an important role in inflammation. PPAR*α* and PPAR*γ* agonists can ameliorate chronic inflammatory conditions, such as atherosclerosis, arthritis, and inflammatory bowel disease [20, 29, 30]. PPARs repress genes of the inflammatory response pathway, such as cytokines (TNF*α*, IL-1*β*, IL-6), cell adhesion molecules (MMPs), and other proinflammatory molecules (iNOS) [25]. These effects are mediated in large part by the ability of PPARs to antagonize other transcription factors, like AP-1, STAT1, and NF-*κ*B, which have proinflammatory functions [25]. Different mechanisms have been proposed to explain the phenomenon of transrepression by PPARs, including sequestration of limiting cofactors, direct physical interaction, and antagonism between PPARs and other transcription factors, and promoter-specific block of corepressor/coactivator exchange by PPARs in selected target genes [24, 25]. The latter involves a block of the ubiquitin and proteasome-dependent processing of corepressor complexes as in the case of PPAR*γ*-mediated repression of proinflammatory NF-*κ*B target genes [25]. PPAR*γ* and PPAR*α* can also interact physically with NF-*κ*B and c-Jun blocking transcriptional activation [33, 34]. Reciprocally, NF-*κ*B and c-Jun can repress PPAR*γ* and PPAR*α*-induced transcription, respectively, by inhibiting the binding to PPRE in target genes [33–35]. Also PPAR*δ* has a role in inflammation controlling expression of proinflammatory genes in macrophages in a ligand-dependent manner [31]. Unliganded PPAR*δ* binds to corepressor molecules including Bcl-6, which is a repressor of inflammatory gene expression [31, 36]. Ligand binding releases the corepressor complexes resulting in transcription of PPAR*δ* target genes. At the same time, PPAR*δ*-bound Bcl-6 is also released and is free to repress its own target genes suppressing the inflammatory response [31, 36]. Paradoxically, PPAR*δ* knockout has the same effects of the agonists on the expression of Bcl-6 target genes since it also leads to release the transcriptional repressor [36].

The involvement of PPARs in carcinogenesis has been widely discussed, although it is still controversial whether the different isotypes either favor or inhibit tumorigenesis [37, 38]. This may still represent a major concern for developing PPAR-targeted therapeutics for clinical applications because of the potential risk of promoting tumorigenesis as indicated by studies in rodents [39]. PPARs are expressed in several human cancers and PPAR ligands have been shown to modulate tumor growth [37, 38]. Inactivating mutations, deletions and chromosomal translocations of PPAR*γ* have been found in various cancers pointing to a tumor suppressor role of this nuclear receptor [40–42]. PPAR*γ* ligands promote differentiation, growth arrest, and death of cancer cells in vitro [38]. PPAR*γ* ligands reduce growth of human tumor xenografts and spontaneous and carcinogen-induced tumors in rodents [38]. PPAR*α* is also expressed in various tumors and cancer cell lines [43, 44]. Activation of PPAR*α* in cancer cells inhibits proliferation and suppresses metastatic potential [45–47]. PPAR*α* ligands have shown antitumor activity also in murine models [46, 48, 49]. PPAR*δ* participates in a number of important pathways controlling adhesion, proliferation, differentiation, and survival [37]. Unlike the other isotypes, PPAR*δ* has been shown to prevent apoptosis and induce cell growth in normal cell types, like primary mouse keratinocytes, preadipocytes, vascular smooth muscle cells, hepatic stellate cells [37]. Consistent with an antiapoptotic role, PPAR*δ* increases the expression of antiapoptotic genes and activates prosurvival signaling pathways in keratinocytes [50]. PPAR*δ* agonists stimulate proliferation and survival of cancer cells in vitro and promote tumor growth in mice [51–57]. PPAR*δ* is a downstream target of *β*-catenin/T cell factor-4, which is central in colon cancer pathogenesis and regulates other cancer-promoting genes like c-myc and cyclin D1 [58]. Cyclooxygenase-2 (Cox-2) modulates PPAR*δ* activity and nonsteroidal anti-inflammatory drugs that have chemopreventive effects in colon cancer inhibit PPAR*δ* activity and expression [58–60]. Cox-2 is frequently upregulated in cancer and preneoplastic lesions, and Cox-2 products like PGI_2_ act as selective agonists of PPAR*δ* [58–60]. To further support a protumorigenic role of PPAR*δ*, PPAR*δ* expression is elevated in cancers, like colorectal, endometrial, and head and neck cancers [58, 59, 61]. Additional evidence pointing to a tumor promoting function of PPAR*δ* comes from experiments in mice where disruption of PPAR*δ* decreased tumorigenicity of cancer cells in nude mice and PPAR*δ* activation increased tumor growth [55, 57, 62].

Despite this large body of evidence, some controversial results in animal experiments cast doubts both on the anti- and protumorigenic activities of PPARs [37, 38]. Experiments in rodents have shown increased frequency and enhanced tumor growth by PPAR*γ* agonists [38, 63, 64]. Similar contradictory data have been reported for PPAR*α*, whereby prolonged administration of PPAR*α* agonists caused hepatocarcinogenesis in rats and mice [65]. The frequency of intestinal tumors also increased in PPAR*δ* knockout mice [66, 67] or decreased upon treatment of the animals with PPAR*δ* ligands [68]. These contradictory results between cellular and animal models and different animal models suggest that the function of these nuclear receptors is more complex than that has been assumed so far and may depend heavily on cell and tissue context, cross-talks with multiple signaling pathways and noncell autonomous mechanisms. A hint to this complexity is given by recent studies of the role of PPARs in tumor angiogenesis. In addition to cancer cell-autonomous effects, PPARs affect strongly tumor angiogenesis and inflammation, two processes that have a critical role in tumor pathogenesis and progression. PPAR*γ* and PPAR*α* agonists have anti-inflammatory properties, which may contribute greatly to their in vivo antitumor activity under certain circumstances. PPAR*γ* ligands are also potent angiogenic inhibitors [69, 70] and PPAR*α* agonists suppress VEGF production, endothelial cell proliferation, and tumor growth in mice [48, 49]. PPAR*δ* activation stimulates VEGF production in mice, which at least in part had an autocrine prosurvival effect on cancer cells [71]. PPAR*δ* has been recently identified as a critical node in a tumor angiogenic network linking angiogenesis to inflammation and carcinogenesis [72]. Knockout of PPAR*δ* in host tissues but not in tumor cells reduced tumor growth by impairing angiogenesis [72]. Interestingly, the in vivo antitumor activity of PPAR*α* agonists also depended heavily on the effects of host endothelial and stromal cells rather than cancer cells blocking angiogenesis and inflammation [48, 49]. Paradoxically, PPAR*α* knockout impaired tumor growth in mice, because it resulted in a strong inflammatory response and production of anti-angiogenic factors, like TSP-1 and endostatin [73]. This paradoxical response is similar to the effects of PPAR*δ* on inflammatory gene expression in macrophages, where both receptor activation and knockout suppressed expression of a subset of target genes [31, 36]. This dual mode of regulation of gene expression, whereby ligand- dependent and independent mechanisms lead to transrepression, derepression, or trans-activation of distinct subsets of genes, seems a common theme for these nuclear receptors and needs to be taken into account when examining their functions in physiological and pathological processes.

## 4. THE UBIQUITIN-PROTEASOME SYSTEM AND PEROXISOME PROLIFERATOR-ACTIVATED RECEPTORS

### 4.1. UPS and control of PPAR turnover

Important factors to consider when studying the multiple and complex functions of PPARs are their connections with other cellular systems and how these interactions reciprocally impact on each system activity. Recent reports suggest that the activity of PPARs is linked in many ways to the UPS [28]. All three PPARs are short-lived proteins that undergo ubiquitination and proteosomal degradation and the UPS is mainly responsible for the turnover of these nuclear receptors [28]. However, the three PPAR isotypes have different behaviors with respect to ligand-dependent receptor turnover. PPAR*γ* undergoes negative autoregulation upon agonist binding. PPAR*γ* is ubiquitinated and degraded by the proteasome in a negative feedback loop that probably serves to attenuate receptor-mediated gene transactivation [74]. PPAR*α* turnover is controlled by ligands in a slightly different manner. Instead of enhancing ubiquitination and degradation, PPAR*α* ligands prevent ubiquitination and lead to increased stability of the receptor [75]. The protective effect of the ligand, however, is maximal during the first 3 hours of exposure to the ligand and the receptor is then rapidly degraded [75].

We have recently examined the ligand-dependent turnover of PPAR*δ* and the role of the UPS in this process [76]. Our study revealed interesting differences between PPAR*δ* and other PPAR isotypes with respect to ligand-dependent receptor turnover and interaction with the UPS. We found that PPAR*δ*, like other nuclear receptors, is ubiquitinated and rapidly degraded by the proteasome [76]. Brief incubation of cells expressing both endogenous and recombinant PPAR*δ* with proteasome inhibitors led to rapid accumulation of the receptor in cell nuclei. Interestingly, in the presence of proteasome inhibitors, PPAR*δ* was transcriptionally competent as shown by luciferase reporter assays and assessment of endogenous target genes by RT-PCR [76]. Thus, PPAR*δ* was different from other nuclear receptors, including the estrogen, androgen, thyroid hormone, and retinoic acid receptors, whose transcriptional activity is reduced by proteasome inhibitors [26]. Furthermore, while in the absence of ligands PPAR*δ* had a very short half life (~30 minutes), the addition of ligand increased considerably the receptor half life [76]. The effects of the synthetic and natural ligands were rapid with an increase of PPAR*δ* protein level within 4 hours upon addition to the cell culture medium. The receptor level remained high as long as the ligands were present [76]. Removal of the ligands was followed by rapid reversal with return to the baseline level within few hours. Once again, PPAR*δ* behavior was unique among nuclear receptors, whose turnover is generally accelerated by their own ligands [26, 77]. The progesterone receptor, thyroid receptor, estrogen receptor, RAR, and RXR all show ligand-dependent increase of degradation associated with transcriptional activation [26, 77]. The direct consequence of these events is a rapid decrease of the receptor half life and switching-off the transcriptional response. Only vitamin D3 receptor is known to be stabilized by the ligand with a similar kinetics [78]. As mentioned above, PPAR*γ* is also rapidly degraded upon exposure to ligands [74] and PPAR*α* is stabilized only transiently by ligands [75]. Further work demonstrated that ligand-induced stabilization of PPAR*δ* was due to a selective block of receptor ubiquitination [76]. This ubiquitination block depended on the continuous presence of the ligand, was rapidly reversed after removal of the ligand, and was due to the direct interaction of the ligand with the receptor [76]. Disruption of the LBD in PPAR*δ*/Tr1-299 abolished the effect of the ligand on ubiquitination and proteolysis, although the truncated form of the receptor was still ubiquitinated and degraded by the proteasome [76]. Thus, binding of the ligand to the LBD induced a conformational change that, in addition to allowing receptor trans-activation, blocked the interaction of PPAR*δ* with an ubiquitin ligase or, alternatively, promoted binding of a deubiquitinating enzyme.

**Figure 2:**
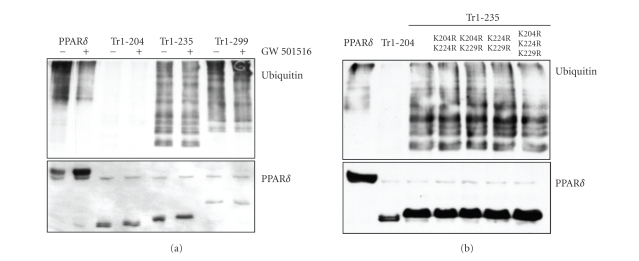
Ubiquitination of truncated and mutated forms of PPAR*δ*. (a) U2OS cells were transfected with HA-ubiquitin expressing vector along with wild type His-PPAR*δ* or truncated forms of the receptor (PPAR*δ*/Tr1-204, Tr1-235 and Tr1-299). After 24 hours, cells were incubated overnight with vehicle or the PPAR*δ* ligand GW501516 (5 *μ*M) and subsequently all samples were incubated with 10 *μ*M the proteasome inhibitor PS341 for 4 hours. His-tagged wild type and truncated PPAR*δ* were pulled-down with nickel affinity gel under denaturing conditions. PPAR*δ* was detected in pull-down fractions using an anti-His antibody and ubiquitinated proteins with an anti-HA antibody. (b) U2OS cells were transfected with HA-ubiquitin vector along with the indicated PPAR*δ* expressing vectors. PPAR*δ*/Tr1-235 had wild type sequence or the indicated double or triple mutations (K204R, K224R and K229R). Cells were treated and analyzed as above.

Using site-directed mutagenesis, we investigated further the role of distinct PPAR*δ* domains in the ligand-dependent regulation of receptor turnover [76]. This analysis revealed additional differences between PPAR*δ* and other PPAR isotypes. Mutations in the DBD of PPAR*δ* reduced the effect of ligands on receptor ubiquitination [76]. This suggested that the ligand acted preferentially on the DNA-bound receptor preventing its ubiquitination. Interestingly, mutations in DBD of PPAR*γ* did not affect ligand-dependent turnover, indicating that DNA binding was not a prerequisite for ligand-induced degradation of this receptor [74]. On the other hand, we showed that the AF-2 domain of PPAR*δ* was not required for ligand-induced block of ubiquitination, indicating that the effect was independent of coactivator binding [76]. For most nuclear receptors, the transactivating function is linked to proteolytic degradation and mutations in the transactivating domain affect also receptor ubiquitination and proteolysis [77]. The AF-2 domain of PPAR*γ* has a similar role and mediates ligand-induced degradation of the receptor [74]. For PPAR*γ* and other nuclear receptors, conformational changes induced by the ligands may favor the concomitant interaction with coactivators and components of the UPS. Overexpression of transcriptional coactivators led also to a decrease of PPAR*α* level in the presence of ligand, showing that the interaction with coactivators via the AF-2 domain promoted proteolysis of the *α* isotype [79]. Thus, for PPAR*α* the initial stabilization is probably followed by the recruitment of coactivators along with other factors that trigger proteolysis of the receptor. In contrast, in the case of PPAR*δ* we showed that transactivation and receptor ubiquitination are functionally separated [76]. The absence of a link between these two processes allows independent control of receptor transactivation and ubiquitination upon ligand binding and may be a prerequisite to avoid rapid degradation and sustain its transcriptional activity once it is engaged in transcriptional activation complexes. Further analysis of PPAR*δ* mutants indicates that the region between amino acid 204 and 235 may play a role in controlling ubiquitination and proteolytic degradation of the receptor (Figure 1). This region has a poor secondary structure, forms a loop exposed to the surface, and may be in an environment prone to ubiquitination [80, 81]. In addition, the region is quite diverse between the PPAR isotypes, possibly explaining the divergent responses in terms of ligand-dependent turnover. Pull-down experiments showed that the truncated PPAR*δ*/Tr1-235 was ubiquitinated, while the shorter PPAR*δ*/Tr1-204 was not (Figure 2(a)). Different scenarios can explain these results and are under consideration. The region between amino acid 204 and 235 may contain lysine residues that are the major sites of ubiquitination of PPAR*δ*. However, mutations of the three lysines present in this region (K204R, K224R and K229R) did not affect ubiquitination of the PPAR*δ*/Tr1-235 (Figure 2(b)). Thus, alternatively the region 204–235 may be needed for the binding of an ubiquitin ligase or cofactors that mediate the interaction of the receptor with the UPS.

Thus, even if the PPAR isotypes are structurally very similar, binding to specific ligands induces divergent responses as far as receptor turnover. PPAR*γ* upon ligand binding becomes ubiquitinated and prone to degradation, whereas ligands prevent or delay ubiquitination and degradation of PPAR*δ* and PPAR*α*. Most nuclear receptors exhibit negative autoregulation upon interaction with the respective ligands [26, 77]. Ligand-induced stabilization is a less common and has been observed only for very few nuclear receptors. The system in place for PPAR*δ* may be geared to prevent both accumulation of high levels of the receptor and its prolonged activation [76]. Overactivity of PPAR*δ* may be detrimental to cells, perhaps due to its antiapoptotic and potentially tumorigenic activity [32, 37]. The level of PPAR*δ* is low and constantly controlled via UPS-dependent proteolysis, which may affect greatly the ligand-independent functions of the receptor like transrepression of other transcription factor target genes. Under physiological conditions, the low abundance and short half life of natural PPAR*δ* ligands, like PGI_2_, would contribute to keep the receptor in the unbound state [32]. In the presence of high concentrations of ligands, the DNA-bound and liganded PPAR*δ* is protected from proteasomal degradation by the inhibition of its ubiquitination [76]. The stabilized DNA-bound receptor would be able to transactivate target genes as long as enough ligand is present. This would be consistent with the fact that in processes, such as wound healing, inflammation, and cancer, PPAR*δ* levels seem to increase concomitantly with upregulation of cyclooxygenase-2 and other enzymes for the production of lipid metabolites capable of stabilizing and activating PPAR*δ* [32, 37, 55, 58, 59]. In the absence of this coordinated increase of ligand and receptor levels, PPAR*δ* might not be able to act as antiapoptotic and growth-promoting factor. How ligand-induced stabilization of PPAR*δ* affects ligand-dependent interactions with other transcription factors leading to transrepression or derepression of gene expression is still unknown.

### 4.2. UPS, PPARs, and interactions with other signaling pathways

**Figure 3:**
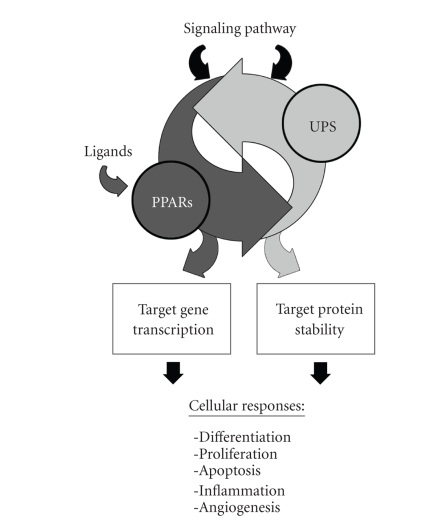
*Interactions between PPARs and the ubiquitin-proteasome system (UPS) affect multiple cellular pathways*. The UPS regulates activity of PPARs by controlling receptor turnover in ligand dependent and independent manners and affecting the ability of PPARs to regulate target gene transcription. Signaling pathways can modulate PPAR activity by affecting UPS-mediated turnover (e.g., increased PPAR*γ* degradation in response to growth factors or hormones). PPAR can also affect biological pathways and cellular responses by increasing or decreasing susceptibility of proteins to proteasomal degradation (e.g., enhanced degradation of *β*-catenin and suppression of the Wnt pathway by PPAR*γ*).

In addition to ligand-dependent receptor turnover, the UPS is an important way to control PPAR activity in response to upstream signal transduction pathways (Figure 3). Receptor phosphorylation by cellular kinases can regulate both basal and ligand-induced activity of PPARs as well as modulate their protein level by indirectly controlling proteasome-dependent degradation [82]. In colorectal cancer cells, the polypeptide hormone gastrin promotes cell proliferation and the effect is associated with decreased PPAR*γ* level. This was mediated by phosphorylation of PPAR*γ* involving the epidermal growth factor receptor and ERK1/2 kinase leading to increased PPAR*γ* proteasome-mediated degradation [83]. In fat cells IFN-*γ* treatment induces a rapid reduction of PPAR*γ* protein level, which is blocked by proteasome inhibitors [84]. On the other hand, there are instances in which PPARs enhance stabilization or degradation of proteins by affecting their susceptibility to UPS-mediated degradation. Perhaps the best example of a signaling pathway in which both PPARs and the UPS are implicated is the Wnt pathway. Suppression of the canonical Wnt signaling is required for differentiation of preadipocytes into adipocytes. The process is in part mediated by PPAR*γ*-induced degradation of *β*-catenin, which is a central element in the Wnt pathway. Activation of PPAR*γ* promotes degradation of *β*-catenin in glycogen synthase kinase 3*β* (GSK3B)-dependent or independent manner [85, 86]. *β*-catenin mutations that inhibit degradation block expression of a subset of adipogenic and PPAR*γ* target genes [85]. PPAR*γ*-dependent degradation of *β*-catenin requires an active APC-containing destruction-complex. Mutations of the T cell factor/lymphocyte enhancer factor (TCF/LEF) binding domain of *β*-catenin or of a catenin-binding domain (CBD) within PPAR*γ* block proteasomal degradation of *β*-catenin [87]. The interaction between *β*-catenin and PPAR*γ* affect their respective oncogenic and tumor suppressor function [87]. A functional APC was found to be required also for PPAR*γ*-mediated suppression of colon carcinogenesis [88]. Activation of PPAR*γ* induces degradation of cyclin D1, which has a critical role in cell cycle regulation, along with *β*-catenin in hepatocytes [89]. Reduced cyclin D1 protein level was observed also in breast cancer cells upon PPAR*γ* activation by selective ligands and cyclin D1 downregulation was blocked by inhibition of the proteasome [90]. However, the ability of thiazolidinedione ligands to reduce *β*-catenin and cyclin D1 levels might be in part PPAR*γ*-independent and determined by direct effects of these compounds on protein degradation [91, 92]. Beside the induction of proteosomal degradation, activation of PPAR*γ* has been shown to increase the level of proteins by blocking their proteolysis. Activation of PPAR*γ* in human hepatocarcinoma cells inhibits proteosomal degradation of p27, a cyclin-dependent kinase inhibitor, with consequent inhibition of cell proliferation [93]. Similarly, PPAR*γ* inhibits claudin 4 degradation resulting in urothelial cell differentiation [94]. In both cases, the increased protein level is probably due to reduced ubiquitination. Interestingly, transcriptome analysis of ovarian cancer cells exposed to a PPAR*γ* agonist revealed that PPAR*γ* activation resulted in upregulation of several genes involved in protein modification and ubiquitination, including many ubiquitin ligases and ubiquitin-conjugating enzymes [95]. This finding may provide a plausible explanation for the broad effects that PPAR-*γ* agonists have on protein ubiquitination and turnover and clearly deserves further investigation [95].

PPAR*α* agonists also enhance protein degradation. In LPS-treated macrophages PPAR*α* agonists enhance degradation of inducible nitric oxide synthase (iNOS), reducing nitric oxide (NO) production, which is an important mediator in inflammatory processes. PPAR*α* agonists did not affect iNOS expression and proteasome inhibitors reversed the effect on iNOS protein levels, indicating that PPAR*α* agonists enhanced degradation of this protein by the proteasome [96]. PPAR*δ* has been found to regulate ubiquitin C expression and this has been linked to the modulation of protein kinase C*α* (PKC*α*) and attenuation of cell proliferation in the skin. The level of PKC*α* was lower in the skin of PPAR*δ* wild-type mice treated with TPA compared to the skin of PPAR*δ*-null mice [97]. On the other hand, the amount of ubiquitinated PKC*α* was lower in skin of TPA-treated PPAR*δ*-null mice compared to wild-type mice and inhibition of the proteasome prevented TPA-induced downregulation of PKC*α*. Thus, the effects of PPAR*δ* on cell proliferation in the skin could be due to ubiquitin-dependent turnover of PKC*α* that in turn modulated the activity of the PKC*α*-dependent pathways [97].

Finally, the UPS is involved in the reciprocal regulation of PPARs and other transcription factors. Activation of NF-*κ*B is achieved when the inhibitor I*κ*B, which normally holds NF-*κ*B in the cytoplasm, is phosphorylated and recognized by the E3-*β*-transducin repeat containing protein (*β*-TRCP). Ubiquitinated I*κ*B is degraded by the proteasome, allowing NF-*κ*B to translocate to the nucleus and induce gene transcription [98]. NF-*κ*B has a critical role in inflammation. In experimental rat models of autoimmune myocarditis stabilization and translocation of NF-*κ*B were inhibited by PPAR*γ*-dependent expression of I*κ*B [99]. Likewise, PPAR*α* activation induced I*κ*B in aortic smooth muscle cells and in human hepatocytes [100]. The transcription factor AP-1, which is another key player in inflammation, interacts with the PPARs and may be regulated in a similar combinatorial manner by PPARs and the UPS [33, 34].

## 5. CONCLUSIONS

Here, we have presented the current evidence linking PPARs and the UPS. Ubiquitination and proteasomal degradation control the level and modulate the activity of PPARs in many ways. Ligand binding and proteolytic degradation affect turnover and transcriptional activity of the PPAR isotypes in distinct ways. PPAR*δ* ubiquitination is selectively blocked by agonist ligands ensuring the accumulation of DNA-bound receptor engaged in transcriptional activation complexes. The opposite is true for the other PPAR isotypes. Distinct cellular pathways can exploit the UPS to modulate PPAR turnover and activity affecting their multiple functions. Furthermore, PPARs can control the level of specific proteins by modulating the activity of the UPS. This could be mediated by their ability to control the expression of components of the UPS, like ubiquitin ligases, or via protein-protein interactions. Controlling turnover of the receptors, the UPS can affect also the ligand-independent functions of PPARs. In this context, the control operated by the UPS on nuclear receptor levels might affect their ability to modulate activity of other transcriptional regulators. Increased proteolysis might reduce PPAR levels and produce apparently paradoxical responses with derepression or transrepression of distinct subsets of genes as seen in certain PPAR knockout experiments. The contribution of the multiple interactions between PPARs and the UPS need to be taken in consideration when examining the effects of PPAR overexpression, knock down or ligand-dependent activation on complex biological processes, like inflammation, angiogenesis, and tumorigenesis.
